# Pretreatment patient-specific quality assurance prediction based on 1D complexity metrics and 3D planning dose: classification, gamma passing rates, and DVH metrics

**DOI:** 10.1186/s13014-023-02376-4

**Published:** 2023-11-20

**Authors:** Liyuan Chen, Huanli Luo, Shi Li, Xia Tan, Bin Feng, Xin Yang, Ying Wang, Fu Jin

**Affiliations:** Department of Radiation Oncology, Chongqing University Cancer Hospital, Chongqing, 400030 China

**Keywords:** Deep learning, Patient-specific quality assurance, Complexity metrics, Dose-volume histograms

## Abstract

**Purpose:**

Highly modulated radiotherapy plans aim to achieve target conformality and spare organs at risk, but the high complexity of the plan may increase the uncertainty of treatment. Thus, patient-specific quality assurance (PSQA) plays a crucial role in ensuring treatment accuracy and providing clinical guidance. This study aims to propose a prediction model based on complexity metrics and patient planning dose for PSQA results.

**Materials and methods:**

Planning dose, measurement-based reconstructed dose and plan complexity metrics of the 687 radiotherapy plans of patients treated in our institution were collected for model establishing. Global gamma passing rate (GPR, 3%/2mm,10% threshold) of 90% was used as QA criterion. Neural architecture models based on Swin-transformer were adapted to process 3D dose and incorporate 1D metrics to predict QA results. The dataset was divided into training (447), validation (90), and testing (150) sets. Evaluation of predictions was performed using mean absolute error (MAE) for GPR, planning target volume (PTV) HI and PTV CI, mean absolute percentage error (MAPE) for PTV D_95_, PTV D_2_ and PTV D_mean_, and the area under the receiver operating characteristic (ROC) curve (AUC) for classification. Furthermore, we also compare the prediction results with other models based on either only 1D or 3D inputs.

**Results:**

In this dataset, 72.8% (500/687) plans passed the pretreatment QA under the criterion. On the testing set, our model achieves the highest performance, with the 1D model slightly surpassing the 3D model. The performance results are as follows (combine, 1D, and 3D transformer): The AUCs are 0.92, 0.88 and 0.86 for QA classification. The MAEs of prediction are 0.039, 0.046, and 0.040 for 3D GPR, 0.018, 0.021, and 0.019 for PTV HI, and 0.075, 0.078, and 0.084 for PTV CI. Specifically, for cases with 3D GPRs greater than 90%, the MAE could achieve 0.020 (combine). The MAPE of prediction is 1.23%, 1.52%, and 1.66% for PTV D_95_, 2.36%, 2.67%, and 2.45% for PTV D_2_, and 1.46%, 1.70%, and 1.71% for PTV D_mean_.

**Conclusion:**

The model based on 1D complexity metrics and 3D planning dose could predict pretreatment PSQA results with high accuracy and the complexity metrics play a leading role in the model. Furthermore, dose-volume metric deviations of PTV could be predicted and more clinically valuable information could be provided.

**Supplementary Information:**

The online version contains supplementary material available at 10.1186/s13014-023-02376-4.

## Introduction

Highly modulated radiation therapy plan offers highly conformal dose distributions to the target volume while maintaining steep dose gradients around the target area. Considering the planning and delivery complexity, patient-specific quality assurance (PSQA) measurements are regularly implemented before the first treatment fraction of intensity modulated radiation therapy (IMRT) and volumetric modulated arc therapy (VMAT) as an important clinical routine [1, 2]. These measurements serve multiple important purposes, including validating dosimetric calculations, confirming accurate data transfer, and ensuring machine deliverability [3]. PSQA typically required the complete delivery of the patient’s treatment plan [4, 5], thus, performing measurements for every patient can be resource-intensive, and the actual benefit of these measurements for certain patients remains unclear [6, 7]. Consequently, in busy healthcare institutions, these measurements are typically conducted outside of regular clinical hours and it may be late and hard to react to failed plans. Therefore, the prediction of pretreatment PSQA results would greatly benefit radiotherapy institutions by quickly identifying treatment plans that may require actual measurements and understanding the potential impact of errors in the delivery.

PSQA commonly employs gamma analysis to quantify the agreement between measured and planned dose distributions [8, 9], and gamma passing rate (GPR) provides information about the overall similarity of the total dose or dose within a region of interests (ROI). The TG 218 report recommends 95% or 90% as tolerance of action limits under the 3%/ 2 mm gamma criterion [2]. Moreover, as clinicians are concerned, dose volume histogram (DVH) metrics can offer a more informative depiction of dose discrepancies for clinical decision [10, 11]. Many studies have shown the relevance of DVH metrics to tumor control and normal tissue sparing [12–14], and the ability of DVH to detect clinically relevant dose errors was also revealed [15]. However, there is still a lack of studies that specifically focus on the prediction of DVH metrics.

Previous researches have explored the use of machine learning techniques to predict the outcomes of PSQA in radiotherapy [16–19]. Several studies have reported QA classification results, achieving a macro area under the curve (AUC) value up to 0.88 [3, 21, 22], These studies highlight the potential of machine learning models in prioritizing treatment plans that would benefit the most from PSQA. Furthermore, for GPR prediction, Valdes et al. demonstrated the potential of machine learning algorithms by utilizing complexity metrics to predict the GPR under a 3%/3mm criterion, with maximum prediction errors smaller than 3%. In a multi-institutional validation study, approximately 86% of the plans had a prediction error smaller than 3.5% [20, 21]. Other studies have investigated the use of deep learning algorithms to predict 2D GPR using flux maps, planar dose distributions, and 3D planning dose distributions [22–26]. Prediction results varied based on the chosen gamma criterion. Generally speaking, under a 3% dose difference criterion, the mean absolute error (MAE) was smaller than 2%. However, there are still some limitations. Firstly, the predictions of GPR were mostly based on 2D GPR, there is a lack of research on more informative 3D GPR. Additionally, the inputs to these models are usually unimodal, only a few researches included partial aperture related plan complexity or volume indices in addition to dose characteristics and get good performance [17, 22]. This suggests that the integration of dose characteristics with 1D metrics is advantageous, however, their study only incorporates a limited set of 1D metrics, indicating the necessity of including more comprehensive measures, such as transfer related plan complexities.

The Swin-Transformer is an innovative deep learning architecture designed to tackle complex vision tasks. It introduces a hierarchical structure where small and efficient transformers are used to process local image patches, and then global transformers are employed to capture long-range dependencies among these patches. In comparison to conventional convolutional neural network, this design allows Swin-Transformer to efficiently handle long-range dependency among various image scales and achieve state-of-the-art performance across a spectrum of medical image analysis tasks [27]. Its attention mechanism also facilitates the capture of intricate dose patterns which may reflect the nuances in dose delivery variability and are well-suited for PSQA prediction.

This is the first study introducing a novel deep learning architecture Swin-transformer to develop a PSQA prediction model that incorporates the planning dose and complexity metrics. In addition to the results of gamma analysis, DVH metrics in target volume are also predicted to provide more comprehensive information for clinical practice. Furthermore, the predictive capabilities of 3D planning dose and 1D complexity metrics were compared.

## Materials and methods

### Data collection

**Fig. 1:**
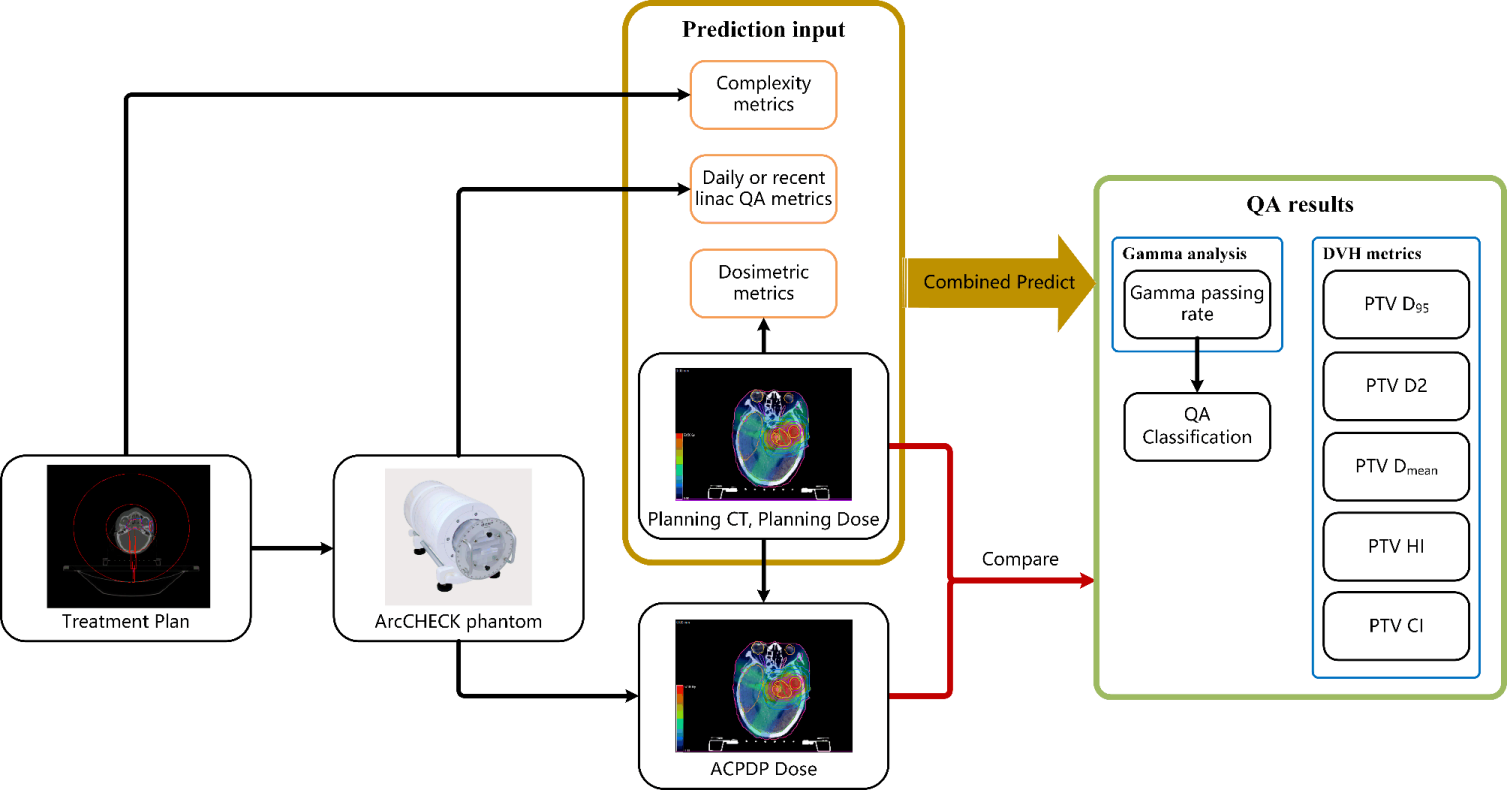
Workflow of this study. The metrics in the orange boxes are utilized as 1D predictive input, while the planning dose is utilized as 3D input to establish 3D only predictive models. And both of them are utilized as multimodal inputs for combined model. ACPDP: ArcCHECK planned dose perturbation algorithm

Patients who received treatment at our institution from March 2022 to April 2023 were included in this study. The patient plans were generated using Eclipse treatment planning system (TPS) (Varian Medical Systems, Palo Alto, CA, version 15.6) or Pinnacle TPS (Philips Radiation Oncology Systems, Fitchburg, WI, version 16.2) and delivered using a Varian IX linear accelerator with a 6MV photon-beam. PSQA was scheduled using the ArcCHECK phantom from Sun Nuclear Corporation (Melbourne, FL, USA). Prior to the study, accelerator commissioning and ArcCHECK phantom configuration were performed following the vendor’s standard procedures. The measurement results obtained from the annular detector matrix of the ArcCHECK phantom were utilized to reconstruct simulated 3D dose distributions on the patient’s CT scans using the planned dose perturbation (PDP) algorithm, which was matched with 3DVH software version 3.0, which is an additional tool to ArcCHECK [28]. The workflow of this research is illustrated in Fig. 1.

Plans with insufficient target coverage resulting from OAR overlap and measurement files with excessive hot or cold points during reconstruction were excluded from the study. Eventually, a total of 687 IMRT and VMAT plan data were successfully reconstructed and used for subsequent model development and verification. Detailed plan information is provided in Table 1. Global GPRs were calculated using a 3%/2mm criterion with a 10% threshold, and a GPR value of 90% was employed to determine QA pass or fail. For DVH metrics, considering the variation in treatment sites among the patients, only metrics of PTV were calculated, which were PTV D_95_ (minimum dose received by 95% of the planning target volume), PTV D_2_, PTV D_mean_ (mean dose received by planning target volume), homogeneity index (HI) and conformity index (CI).

**Table 1:** Plan characteristic distributions

Characteristics	Plan number (%)
**Plan type**	
IMRT	298 (43.4%)
VMAT	389 (56.6%)
**Treatment site**	
Head and neck	182 (26.5%)
Chest and breast	161 (23.4%)
Pelvic cavity and Abdomen	344 (50.1%)
**Gamma passing rate**	
60 − 80%	95 (13.8%)
80 − 90%	92 (13.4%)
90 − 95%	120 (17.5%)
95 − 100%	380 (55.3%)

A total of 687 plans were utilized to calculate the complexity metrics, following the methodology described in previous studies [29, 30]. These complexity metrics encompassed information regarding the machine unit, leaf aperture, and leaf movement. Regular QA for linear accelerators (linacs) is performed as reported in TG142 [31], and the linac QA metrics on or closest to the measuring day were also recorded, such as absolute dose variation, flatness and symmetry. Additionally, certain dosimetric parameters of the plan were considered in the model, including the HI and CI of the target volume, the volume of the PTV, and the prescription dose. Thus, a total of 71 one-dimensional metrics were incorporated into the model, and the specific metrics are outlined in Supplementary Materials Table S1.

### Combined model establishing

**Fig. 2:**
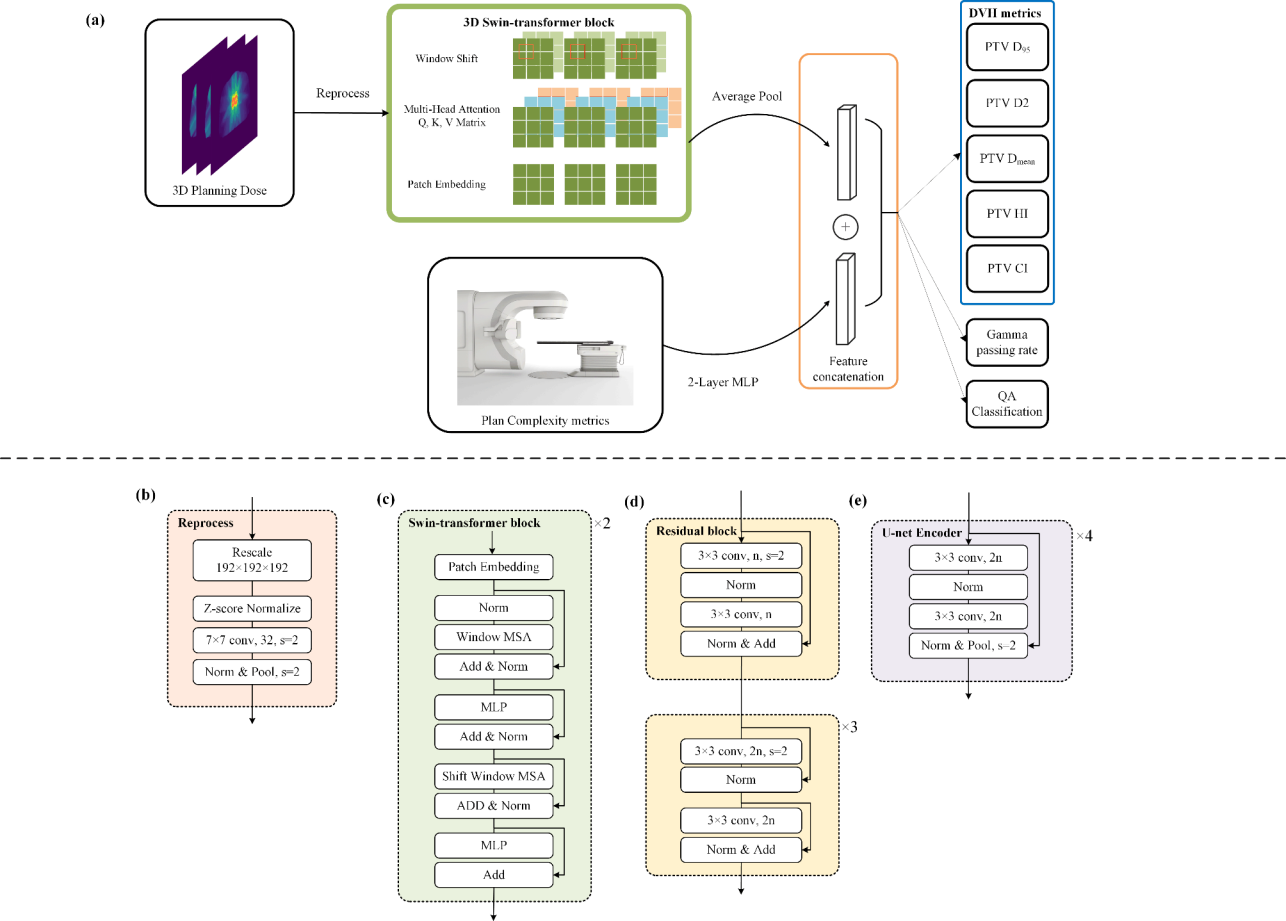
Network architecture utilized in this study. (**a**) The overall architecture of the combined model. (**b**) Reprocess block of the input image, including rescale and a convolution layer for all backbones. (**c**) The simplified Swin-transformer block of 3D version, consisting of two successive layers. (**d**), (**e**) The 3D residual blocks and U-net Encoder utilized in this study for comparison. The number of n or 2n means output channels of the convolution layer and n is the input channel of the block, the s means stride of the convolution kernel or pooling kernel. Norm: Normalization layer, in (b), (d) and (e) means Batch Normalization, in (c) means Layer Normalization. MSA: Multi-head Self-attention. MLP: Multilayer Perceptron

Given the diverse treatment sites of the patients, the sizes of the planning dose grid varied within the range of 63 × 68 × 62 to 336 × 259 × 263. Additionally, there were two sizes of grid spacing: 2.5 × 2.5 × 2.5 mm^3^ and 3 × 3 × 3 mm^3^. To ensure consistency and facilitate analysis, the dose data underwent preprocessing and scaling, resulting in a standardized size of 192 × 192 × 192 before entering the model. As for the 71 1D metrics, normalization was applied using the Z-score method, enabling effective concatenation within the model. In this study, we develop a novel combined architecture based on the Swin-transformer to effectively fuse multimodal inputs [32]. We employed 2 Swin-Transformer blocks, with 4 and 8 heads incorporated in each block. The 3D Swin-transformer block was employed to process the planning doses of patients. Subsequently, the extracted features from the 3D dose were subjected to an average pooling layer, resulting in a one-dimensional feature vector with 256 elements. On the other side, 71 1D inputs were processed through a multi-layer perceptron (MLP) with a hidden layer of 128 units to obtain another 256 1D features. These two types of features were then combined and directly used for multi-task prediction. In the Swin-Transformer block, the MLP was configured with 2 hidden layers, each having 2 times the input dimension units. Dropout layers were incorporated into the model in both 1D metrics processing and Swin-Transformer blocks to prevent overfitting. The architecture of the network employed in this study is illustrated in Fig. 2.

The dataset was split into train, validation and testing. The validation dataset was utilized to fine-tune the hyperparameters of the model during the training phase, and the independent test set was used only once for testing after the model was developed. To assess the regression model’s performance in predicting DVH metrics and GPR, the mean square error (MSE) loss function was employed. For QA classification task, the binary cross-entropy (BCE) loss function was utilized. The overall loss function was defined as the sum of these three individual losses, as depicted in Eq. 1.

1 $$\begin{array}{l}Total\,loss = \frac{1}{N}\sum\limits_{i = 1}^N {{{(\gamma - \hat \gamma )}^2}} + \frac{1}{N}\sum\limits_{i = 1}^N {\sum\limits_{j = 1}^n {{{(D - \hat D)}^2}} } \\\,\,\,\,\,\,\, - \frac{1}{N}\sum\limits_{i = 1}^N ( {y^i}{\rm{log}}{{\hat y}^i} + (1 - {y^i}){\rm{log}}(1 - {{\hat y}^i}))\end{array}$$

Note: N: total number of samples, n: number of DVH metrics, γ: gamma passing rate, D: DVH metrics, y: the probability of passing the QA criteria, means the label of the variable.

Finally, we employed the determined optimal hyperparameters to train the model using a combined dataset consisting of the 447 training samples and 90 validation samples and the performance of the model was evaluated on the independent test set comprising 150 cases. The evaluation of predictions was based on the MAE and the AUC of ROC curve. The proposed deep network architecture was implemented using PyTorch [33] and executed on a NVIDIA GeForce RTX 3090Ti GPU with 24GB memory.

### Other models

Additionally, we also calculated and compared the results obtained using either the 3D dose or the 1D metrics alone (shown in Fig. 1) to assess the individual predictive capabilities of these components as well as the combined model. For 3D model, we adopted commonly used network models in medical dose process, such as ResNet and U-Net encoder, the architectures are shown in Fig. 2. And for 1D model based on 1D metrics, we adopted a three-layer MLP with 2 hidden layers of 128 and 256 units. These training processes were on the same dataset as before. For clinical practice, the results of predicting QA classification directly or determining by predicting GPR were also compared using sensitivity and specificity.

## Results

### Hyperparameters determination

After conducting a search for optimal hyperparameters on a dedicated validation set, we determined that the initial learning rate for the 3D Swin-transformer parameters should be set to 3 × 10^− 7^ and RMSProp (root mean square propagation) optimizer was used. For the remaining parameters of linear layers, the initial learning rate was set to 3 × 10^− 6^. As the training progressed, both learning rates decayed with a dropping rate of 0.98 per 4 epochs. The weight decays of the parameters were set to 10^− 6^ for the 3D Swin-transformer and 10^− 5^ for the other parameters. The Swin-transformer and MLP were trained simultaneously. To train the prediction model, we utilized a batch size of 4, a dropout rate of 0.2, and an epoch number of 200. Subsequently, these hyperparameters were employed to build the prediction model using the 537 cases from the training and validation sets.

### GPR and DVH metrics prediction

**Fig. 3:**
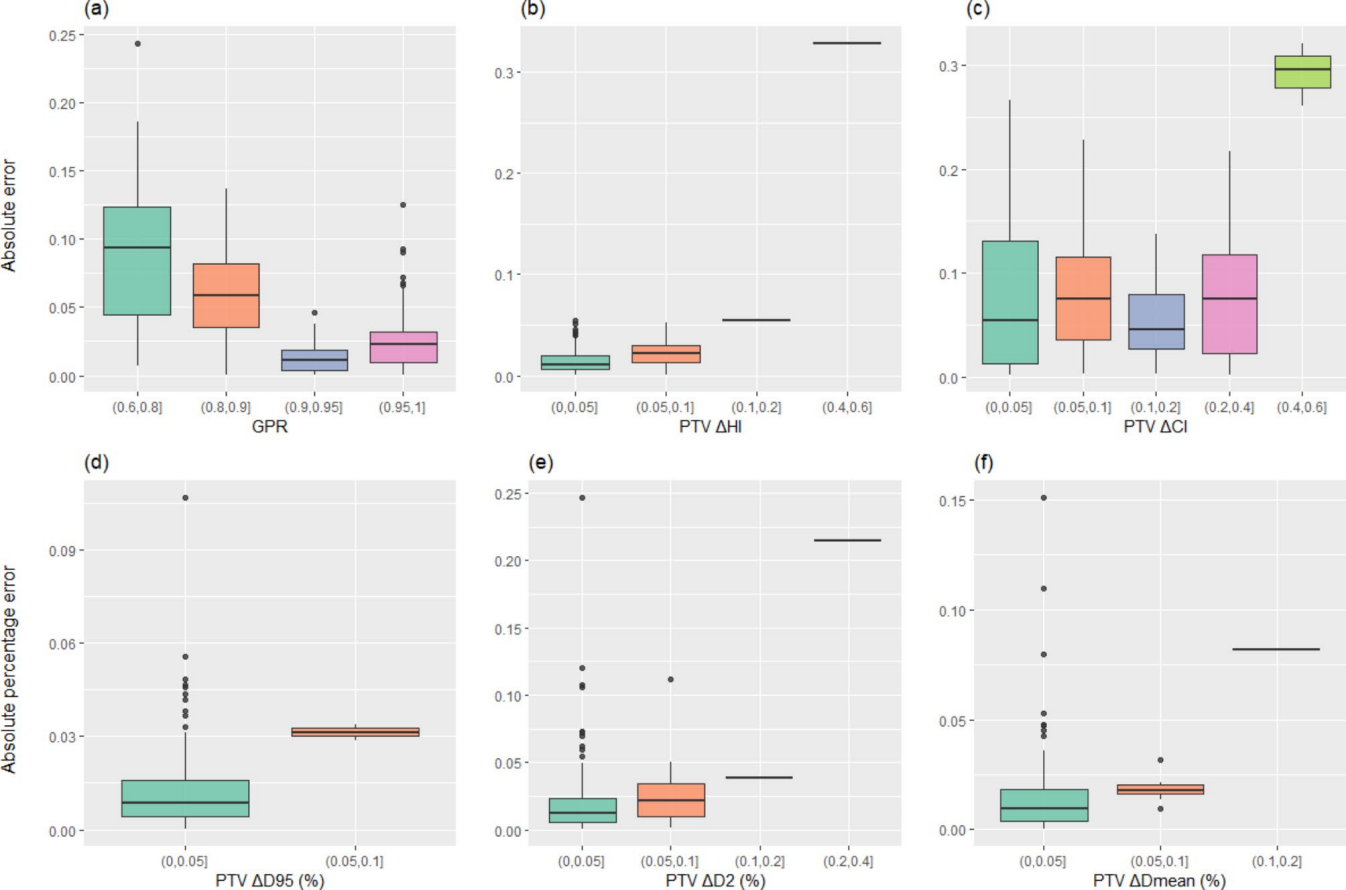
The distribution of absolute errors or absolute percentage errors between the predicted metrics by combined model and the ground truth values for (**a**) GPRs, (**b**) PTV HI, (**c**)PTV CI, (**d**) PTV D_95_, (**e**) PTV D_2_ and (**f**) PTV D_mean_ across different ranges of comparisons between TPS calculation results and ground truth values

Figure 3 illustrates the distribution of absolute errors or absolute percentage errors in the predicted 3D GPRs and the DVH metrics across different ranges of TPS deviations of combined model. The regions characterized by higher GPRs and smaller absolute deviations in DVH metrics display higher prediction accuracy. 60% (90/150) of the plans exhibited predicted percent deviations of PTV D95 within 1%, and 49% (73/150) of the plans demonstrated predicted GPRs with errors within 2%. In the supplementary materials, Figure S.1-S.3 showed the result distributions of other models. Furthermore, Table 2 presents the comparison the performance of various models. The 1D metrics exhibited superior predictive capabilities for GPR and DVH metrics compared to using 3D dose alone. Notably, the model that combined multiple modalities, including 3D dose and plan complexity metrics, achieved the highest prediction accuracy.

**Table 2:** Evaluation indicators of various predictive models with various inputs

		TPS deviation	Combined Model	1D Model	3D Model (Transformer)	3D Model (Res-Net)	3D Model (U-Net)
MAPE (%)	PTV D_95_	2.55	1.23	1.52	1.66	1.69	2.13
	PTV D_2_	3.67	2.36	2.67	2.45	2.62	2.73
	PTV D_mean_	2.36	1.46	1.70	1.71	1.85	2.12
MAE	GPR	/	0.039	0.046	0.040	0.040	0.044
	PTV HI	0.041	0.018	0.021	0.019	0.020	0.019
	PTV CI	0.125	0.075	0.078	0.084	0.094	0.091
AUC	Classfication	/	0.92	0.88	0.86	0.85	0.81
Number of parameters		/	1.9 M	44 K	1.9 M	3.6 M	3.5 M

Note: MAPE: mean absolute percentage error, MAE: mean absolute error, AUC: area under the receiver operator characteristic curve, TPS deviation: ground truth deviations between DVH metrics calculated by TPS and measure-based QA tools

### QA classification

**Fig. 4:**
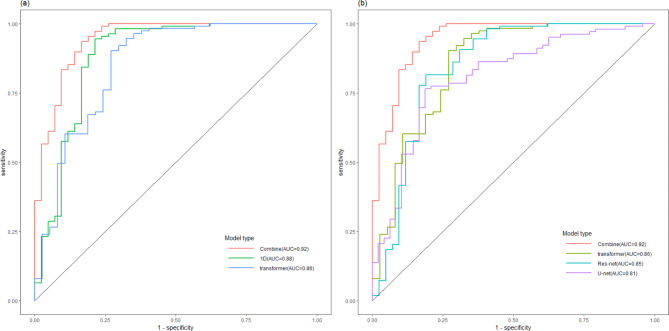
The ROC curves of the QA classification results obtained from various methods. (**a**) Comparison of 1D and 3D inputs. (**b**) Comparison of various models based on 3D input

Figure 4 illustrates the ROC curves of the QA classification results obtained using various models. The corresponding AUC values were 0.92, 0.88, and 0.86 for combined model, 1D model and 3D transformer model respectively. For the QA classification performed using the combined model, the sensitivity was 0.93, and the specificity was 0.92, indicating a high classification accuracy. On the other hand, when classifying based on the predicted GPRs (also 90% as QA criterion), the sensitivity was 0.83, and the specificity was 0.94. The confusion matrices of the classification results in both ways are listed in Supplementary Table S2.

## Discussion

In this study, we developed a transformer-based deep learning prediction model for PSQA by utilizing patient multimodal input including 3D planning dose and 1D complexity metrics parameters. The model demonstrated high accuracy in predicting DVH metrics of PTV, QA classification, and 3D GPR on an independent test dataset. Furthermore, the model showed promising applicability across various treatment sites and treatment technologies. The predictive capabilities of 3D and 1D inputs were also compared.

Measurement-based PSQA remains a crucial step in the clinical workflow of radiotherapy, serving to validate data transfer and ensure the consistency between linac delivery and TPS calculations. Typically, the widely adopted evaluation method for PSQA is gamma analysis, this prediction of PSQA is feasible, but the clinical value is still controversial [34]. And in this context, additional dosimetric analysis based on DVH metrics or dose distributions [35] holds the potential to offer enhanced guidance for clinical practice. In our study, our combined model accurately predicts several crucial DVH metrics of the PTV. However, we did not propose specific action levels for PSQA based on deviations of DVH metrics due to variations in PTV across different treatment sites. While a methodology based on patient populations may be a feasible solution for threshold establishment [36], and it may be more suitable for single-site treatment scenarios.

The complexity of the plan is considered to be associated with delivery accuracy, where lower plan complexity indicates a higher likelihood of accurate delivery [37]. Our study also shows that plan complexity metrics have the ability to predict QA results. Moreover, the predictive performance of complexity metrics surpasses that of using patient planning dose alone, with the combined model incorporating both dose and complexity metrics achieving the highest accuracy. To assess the significance of each 1D metrics, we employed all 1D metrics to train a random forest classification model. The importance of each metric was then calculated using the SHAP (SHapley Additive exPlanations) method [38] The results are presented in Supplementary Figure S4. Among these metrics, the top five important ones were identified as follows: CI, LS_mean_ (Mean value of leaf speed), APLS_S2.0−2.4_ (The average proportion of leaf speed from a given range 2.0-2.4), LA_mean_ (Mean value of leaf acceleration) and SYM_Y_ (Symmetry in Y direction). This indicates that the planning conformity and the complexity of MLC motion significantly impact QA outcomes. These findings can provide guidance of input choice for the development of future QA prediction models.

An important objective of a QA predictive model is to determine whether a treatment plan will pass or fail the QA procedure, thereby assisting the clinical workflow, reducing physicists’ workload. For QA classification, studies showed that direct classification models exhibit higher sensitivity than models classified based on GPR prediction, it is consistent with our results. In clinical PSQA procedure, sensitivity is often prioritized over specificity, as predicting the passing of a heavily biased plan can result in higher clinical costs. Li et al.‘s study achieved 100% (19/19) sensitivity and 87.71% (207/236) specificity in their random forest model during cross-validation [39], the distributions of positive and negative samples imbalanced greatly. While Granville et al.‘s multi-classification model based on mean dose error achieved a macro-AUC of 0.88 using a self-defined classification method [3]. Our study adopts the standard recommended by TG218, which is consistent with our institution’s QA process, and achieves a sensitivity of 0.93 (39/42) in an independent 150 test dataset, considering failures constitute approximately 30% of the total samples.

One limitation of our study is that we focused on predicting only 5 critical DVH metrics of the PTV. However, different institutions may prioritize different DVH metrics for the PTV in different treatment sites. Additionally, the prediction of DVH metrics for OARs is also important in clinical practice. However, due to the inclusion of patients with multiple treatment sites in our study, obtaining the same OAR group is challenging. In the future, our single-site studies will try to address the prediction of DVH metrics of OARs. Another limitation is that we solely focused on the 3%/2mm gamma criterion for evaluating the results. This choice was based on our clinical routine and the distribution of classification results in our dataset, but other criteria were also reported useful and might be considered.

## Conclusions

In this study, we developed a transformer-based deep learning model for patient-specific QA using complexity metrics and 3D dose distributions. The accurate prediction of QA classification and the PTV DVH metrics provides valuable guidance for clinical practice. Our findings demonstrate that complexity metrics play a leading role in the predictive capability of the model, surpassing the predictive performance of 3D dose in this task.

### Electronic supplementary material

Below is the link to the electronic supplementary material.

Supplementary Material 1

## Data Availability

The data that support the findings of this study are available from the corresponding author, upon reasonable request.

## List of abbreviations

AUC: Area under the curve
BCE: Binary cross-entropy
CI: Conformity index
DVH: Dose volume histogram
GPR: Gamma passing rate
HI: Homogeneity index
IMRT: Intensity modulated radiation therapy
Linac: Linear accelerator
MAE: Mean absolute error
MAPE: Mean absolute percentage error
MLP: Multi-layer perceptron
MSE: Mean square error
PDP: Planned dose perturbation
PSQA: Patient specific quality assurance
PTV: Planning target volume
ROC: Receiver operating characteristic curve
ROI: Region of interests
TPS: Treatment planning system
VMAT: Volumetric modulated arc therapy
