# Supplementary material for: Pretreatment patient-specific quality assurance prediction based on 1D complexity metrics and 3D planning dose: classification, gamma passing rates, and DVH metrics
Source: Radiat Oncol. 2023 Nov 20;18:192. doi: 10.1186/s13014-023-02376-4 (PMC10662260; doi:10.1186/s13014-023-02376-4)
Supplement: Supplementary file 1 — Supplementary Material 1 [file 13014_2023_2376_MOESM1_ESM.docx]

**Supplementary Materials**

Table S.1. Complexity metrics, linac QA metrics and dosimetric parameters utilized in this study

| **Number** | **Metrics** | **Full name** |
| --- | --- | --- |
| 1 | MU | Monitor Unit |
| 2 | MU/Gy | Monitor unit per Gy |
| 3 | PMU | Plan normalized monitor unit |
| 4 | PI | Plan averaged beam irregularity |
| 5 | PM | Plan averaged beam modulation |
| 6 | AAV_k_ | Aperture area variability |
| 7 | LSV_k_ | Leaf sequence variability |
| 8 | MCS | Modulation complexity score (combination of LSV and AAV) |
| 9 | LT_average_ | Leaf travel: the average distance traveled by the MLC leaves |
| 10 | LT/MU | Leaf travel per MU (mm/MU) |
| 11 | LS_min_ | Minimum value of leaf speed of MLC |
| 12 | LS_max_ | Maximum value of leaf speed of MLC |
| 13 | LS_mean_ | Mean value of leaf speed of MLC |
| 14 | MIs k=0.2 | Modulation index for speed of MLC k=0.2 |
| 15 | MIs k=0.5 | Modulation index for speed of MLC k=0.5 |
| 16 | MIs k=1.0 | Modulation index for speed of MLC k=1.0 |
| 17 | MIs k=2.0 | Modulation index for speed of MLC k=2.0 |
| 18 | APLS_S0-0.4_ | The average proportion of leaf speed from a given range (S_0-0.4_) |
| 19 | APLS_S0.4-0.8_ | The average proportion of leaf speed from a given range (S_0.4-0.8_) |
| 20 | APLS_S0.8-1.2_ | The average proportion of leaf speed from a given range (S_0.8-1.2_) |
| 21 | APLS_S1.2-1.6_ | The average proportion of leaf speed from a given range (S_1.2-1.6_) |
| 22 | APLS_S1.6-2.0_ | The average proportion of leaf speed from a given range (S_1.6-2.0_) |
| 23 | APLS_S2.0-2.4_ | The average proportion of leaf speed from a given range (S_2.0-2.4_) |
| 24 | APLS_S2.4-2.8_ | The average proportion of leaf speed from a given range (S_2.4-2.8_) |
| 25 | APLS_S2.8-3.2_ | The average proportion of leaf speed from a given range (S_2.8-3.2_) |
| 26 | APLS_S3.2-3.6_ | The average proportion of leaf speed from a given range (S_3.2-3.6_) |
| 27 | APLS_S3.6-4.0_ | The average proportion of leaf speed from a given range (S_3.6-4.0_) |
| 28 | APLS_S4.0-4.4_ | The average proportion of leaf speed from a given range (S_4.0-4.4_) |
| 29 | LA_min_ | Minimum value of leaf acceleration |
| 30 | LA_max_ | Maximum value of leaf acceleration |
| 31 | LA_mean_ | Mean value of leaf acceleration |
| 32 | MIa k=0.2 | Modulation index for acceleration of MLC k=0.2 |
| 33 | MIa k=0.5 | Modulation index for acceleration of MLC k=0.5 |
| 34 | MIa k=1.0 | Modulation index for acceleration of MLC k=1.0 |
| 35 | MIa k=2.0 | Modulation index for acceleration of MLC k=2.0 |
| 36 | APLA_A0-1_ | The average proportion of leaf acceleration from a given range (A_0-1_) |
| 37 | APLA_A1-2_ | The average proportion of leaf acceleration from a given range (A_1-2_) |
| 38 | APLA_A2-3_ | The average proportion of leaf acceleration from a given range (A_2-3_) |
| 39 | APLA_A3-4_ | The average proportion of leaf acceleration from a given range (A_3-4_) |
| 40 | APLA_A4-5_ | The average proportion of leaf acceleration from a given range (A_4-5_) |
| 41 | APLA_A5-6_ | The average proportion of leaf acceleration from a given range (A_5-6_) |
| 42 | APLA_A6-7_ | The average proportion of leaf acceleration from a given range (A_6-7_) |
| 43 | APLA_A7-8_ | The average proportion of leaf acceleration from a given range (A_7-8_) |
| 44 | APLA_A8-9_ | The average proportion of leaf acceleration from a given range (A_8-9_) |
| 45 | APLA_A9-10_ | The average proportion of leaf acceleration from a given range (A_9-10_) |
| 46 | ALG | Average leaf gap |
| 47 | %SA < 5×5 cm^2^ | Percentage of CPs with segment area per CP < 5×5 cm^2^ |
| 48 | PA | PA: Plan average beam area |
| 49 | SAS (1) | Small aperture score 1mm |
| 50 | SAS (2) | Small aperture score 2mm |
| 51 | SAS (5) | Small aperture score 5mm |
| 52 | SAS (10) | Small aperture score 10mm |
| 53 | SAS (15) | Small aperture score 15mm |
| 54 | SAS (20) | Small aperture score 20mm |
| 55 | CLS | Closed leaf score |
| 56 | CAS | Cross-axis score |
| 57 | MAD | Mean asymmetry distance |
| 58 | MD | Modulation degree |
| 59 | C/A | Circumference/area |
| 60 | EM | Edge metric |
| 61 | EAM | Edge area metric |
| 62 | CAM | Converted aperture metric |
| 63 | HI | Homogeneity index ($\frac{\left( D_{2}-D_{98} \right)}{D_{Rx}}$) |
| 64 | CI | Conformity index ($V_{Rx,PTV}^{2}/(V_{Rx}*V_{PTV})$) |
| 65 | Dose_rx_ | Prescription dose of the plan (Gy) |
| 66 | Volume_PTV_ | Size of the PTV (mm^3^) |
| 67 | ABS | Linac absolute dose variations (%) |
| 68 | FLAT_X_ | Flatness in X direction |
| 69 | FLAT_Y_ | Flatness in Y direction |
| 70 | SYM_X_ | Symmetry in X direction |
| 71 | SYM_Y_ | Symmetry in Y direction |

Note: D_n_: minimum dose received by n% of the volume of the ROI, D_Rx_: prescription dose, V_Rx,PTV_: the volume within the PTV that receives the prescribed dose, V_Rx_: the volume within the whole body that receives the prescribed dose, V_PTV_: the volume of the PTV.

Table S.2. The confusion matrices of classification by combined model directly and by predicted GPR of combined model.

|  | **Predicted classification** | | | **Classification by predicted GPR** | | |  |
| --- | --- | --- | --- | --- | --- | --- | --- |
|  |  | Pass | Fail |  | Pass | Fail | Total |
| **Ground truth** | Pass | 99 | 9 | Pass | 102 | 6 | 108 |
|  | Fail | 3 | 39 | Fail | 7 | 35 | 42 |


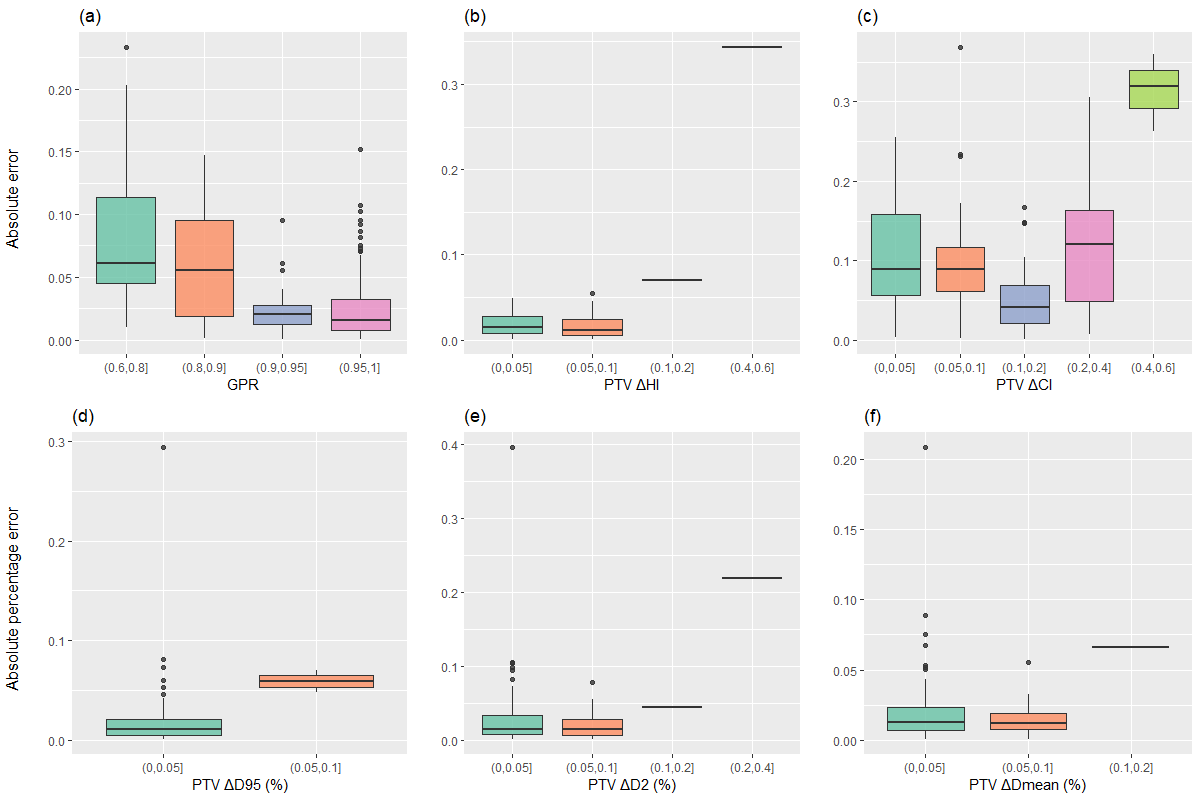


Figure S.1. The distribution of absolute errors or absolute percentage errors between the predicted metrics and the ground truth values across different ranges of comparisons between TPS calculation results and ground truth values for (a) GPRs, (b) PTV HI, (c)PTV CI, (d) PTV D95, (e) PTV D2 and (f) PTV Dmean for 3D swin-transformer model.
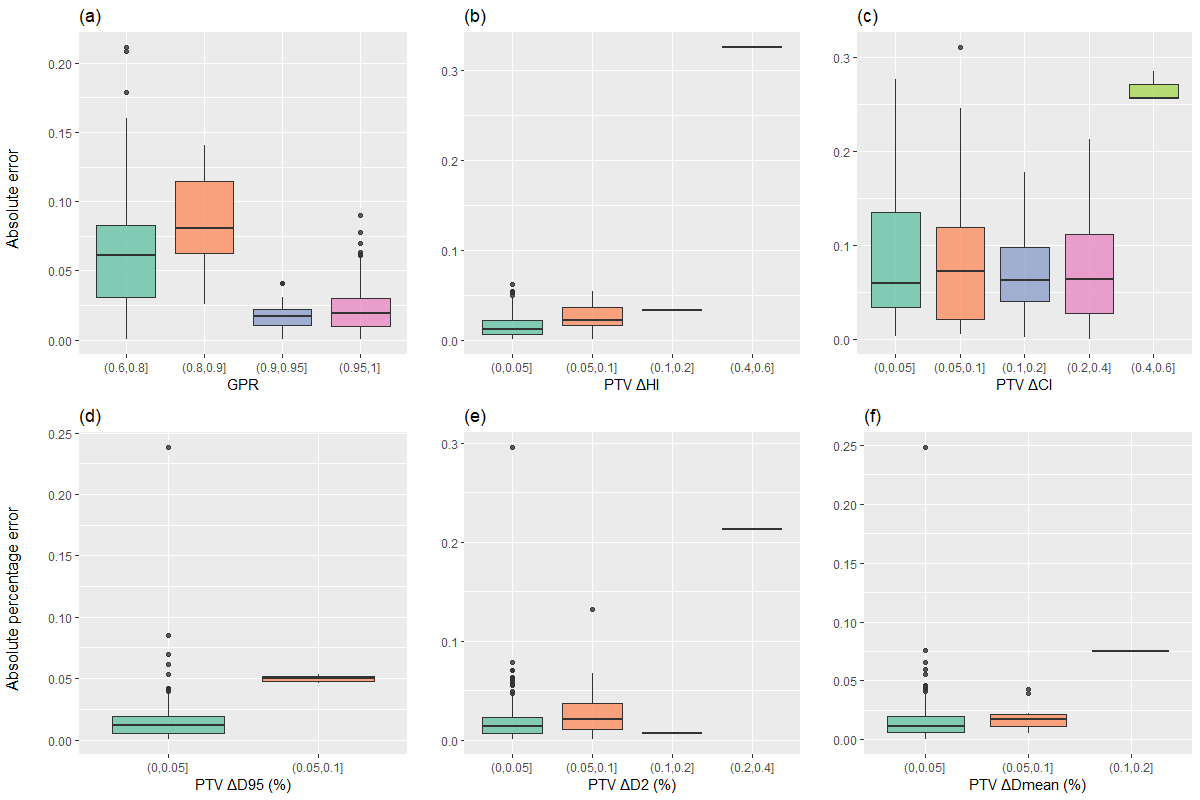


Figure S.2. The distributions for 3D ResNet-18 model.


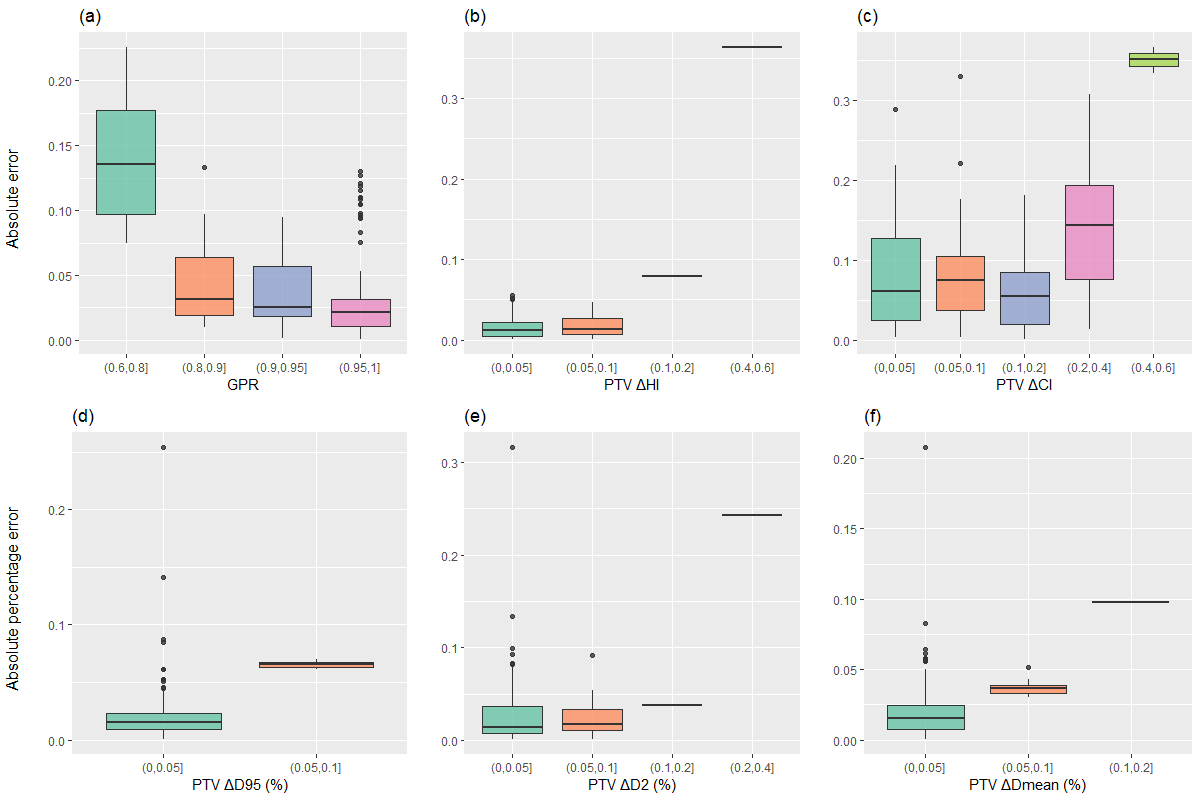


Figure S.3. The distributions for 3D U-Net encoder model.


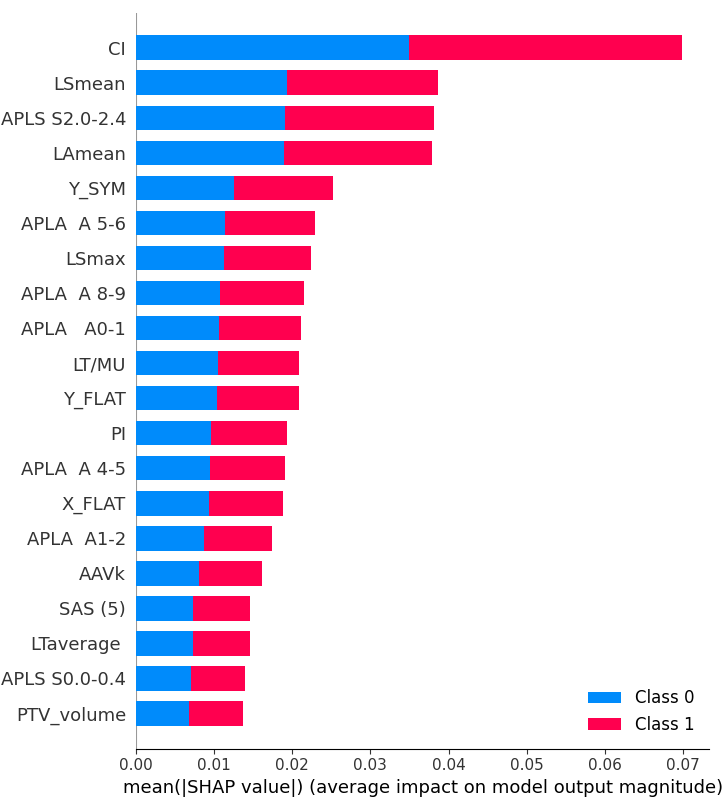


Figure S.4. The importance of each metrics in random forest classification model from shapley additive explanations. See abbreviations in Table S.1.
